# A scoping review of foot and ankle telemedicine guidelines

**DOI:** 10.1002/hsr2.1076

**Published:** 2023-01-20

**Authors:** Lisa Ann Stojmanovski Mercieca, Cynthia Formosa, Nachiappan Chockalingam

**Affiliations:** ^1^ Podiatry Department, Faculty of Health Sciences University of Malta Msida Malta; ^2^ Faculty of Health Sciences University of Malta Msida Malta; ^3^ Centre for Biomechanics and Rehabilitation Technologies Staffordshire University Stoke‐on‐Trent UK

**Keywords:** foot health, guidelines, primary care, telemedicine

## Abstract

**Background and Aims:**

The COVID‐19 pandemic accelerated the adoption of telemedicine in general. Its use has been widely adopted in the healthcare sector, but relatively little research has been conducted on the use of telemedicine for podiatry. This review aimed to explore and compare existing guidelines on telemedicine related to foot and ankle pathologies within a primary care setting.

**Methods:**

The preferred reporting guidelines for the extension of scoping reviews were used in this review, and a set of inclusion and exclusion criteria were developed and implemented. This study made use of both databases and gray literature searches. Between 2012 and 2022, these databases were searched using various subject headings and free‐text terms for the keywords “telemedicine” “foot health” and “guidelines” with appropriate Boolean operators.

**Results:**

The search yielded 356 articles, which were reduced to 283 after removing duplicates. Six more records were discovered through a Google and Google Scholar search and one through an article reference search. Six articles and three institutional practice guidelines were selected for synthesis after screening. The findings were classified according to the level of evidence and research quality, the function of telemedicine and the communication used, the research outcomes sought, and the type of recommendations and guidelines made available.

**Conclusion:**

This review highlights the lack of podiatric telemedicine guidelines for foot and ankle pathologies. Although foot and ankle guidelines for orthopedic and musculoskeletal virtual consultations have been mentioned, they do not cover the full range of potential case scenarios that fall within the remit of podiatric consultations in a primary care setting. This review suggests the development of foot and ankle telemedicine guidelines with recommendations on how they can better provide accessible care to their patients, making foot and ankle care management not only a hand‐on‐one but also reachable virtually, where applicable.

## 1 INTRODUCTION

The World Health Organization defines *telemedicine* as “the provision of healthcare services at a distance with communication between healthcare providers seeking clinical guidance and support from other healthcare providers (provider‐to‐provider telemedicine) or between remote healthcare users seeking health services and healthcare providers (client‐to‐provider telemedicine).“^1^ Over time, the terms “telemedicine,” “telehealth,” “eHealth,” “teleconsultations,” and “remote consultations” were defined separately. However, they are frequently used interchangeably, with their scopes overlapping.^1^

Telemedicine is not a brand‐new concept.^2^ Technological advances have vastly improved telemedicine.^3^ To ensure that healthcare service quality and accessibility are not compromised, the unprecedented crisis caused by COVID‐19 has acted as a catalyst for integrating telemedicine within various healthcare and medical practices.^4^ Transitioning from clinical visits to virtual consultations may have been initially challenging for some healthcare professionals and patients. Nevertheless, many public health institutions worldwide had no choice but to contain the spread of COVID‐19 and better allocate clinical resources. Yet, before any telemedicine service implementation, policymakers and clinicians must be aware of telemedicine's advantages and disadvantages.^5^ Telemedicine offers convenience and better accessibility to healthcare.^4^ Clinical decisions regarding telemedicine use are supported by evidence comparing the clinical effectiveness of standard care and telemedicine service models.^6^ Telemedicine practice guidelines cannot be universal. Telemedicine modalities have been widely utilized in general practice and other fields such as dermatology and psychiatry; however, there are no telemedicine guidelines that cover a broad spectrum of potential foot and ankle conditions for clinicians within this field of practice, such as podiatry. Research for telemedicine application has been conducted on diabetic foot,^7^, ^8^ wound management,^9^, ^10^ and musculoskeletal injuries and complications,^11^, ^12^ but not explicitly related to podiatry consultations, especially at a primary care level.

For telemedicine to be effective during and after COVID‐19 and future events of a similar nature, it must be integrated into health services alongside formalized guidelines, policies, or regulations allowing the implementation of telemedicine practice.^13^ Guidelines educate clinicians on the benefits and drawbacks of telehealth and equip them with evidence‐based recommendations regarding the best, most efficient, and safe methods for providing patient care via telemedicine.^14^

This scoping review is important because it is part of a wider study which is being conducted to develop telemedicine practice guidelines for podiatrists delivering core podiatry within a primary setting. Therefore, this review aimed to identify the most recent telemedicine practice guidelines for the foot and ankle, focusing on guidelines applicable to primary care and to compare these existing guidelines for their content and rigour.

## 2 METHODS

This review followed the preferred reporting guidelines extension for scoping reviews PRISMA‐ScR^15^ (Supporting Information: PRISMA‐ ScR Checklist). The inclusion and exclusion criteria for the systematic search are detailed below.

### 2.1 Patient and public involvement

There was no patient or public involvement as this is a review of already published studies.

### 2.2 Search strategy

This study used a combination of databases and gray literature searches. The paper includes (1) articles found through databases, referred to as articles and (2) guidelines related to the foot and ankle through international professional bodies or their websites, referred to as guidelines. The gray literature search was restricted to guidelines provided by foot health‐related international professional bodies or available on their websites.

PubMed, MEDLINE, Cochrane Central Register of Controlled Trials, and CINAHL databases identified articles through a systematic literature search. Various subject headings and free‐text terms for the keywords “telemedicine,” “foot health,” and “guidelines” with appropriate Boolean operators were used to search these databases between 2012 and 2022. A detailed approach to the search strategy is shown in Supporting Information: Appendix 1.

A Google and Google scholar search were also conducted, and screening for eligible articles and guidelines stopped at page 5, following a similar method used by Godin et al.^16^ Supporting Information: Appendix 2 illustrates the search strategy.

The database search results were imported into the citation management system RefWorks (ProQuest LLC). This software made it possible to eliminate all duplicates. The principal researcher independently assessed the eligibility of the records (titles and abstracts) for inclusion in the study based on the inclusion and exclusion criteria. Subsequently, the principal researcher screened the full texts of the articles and chose those pertinent to the data retrieval procedure. Discussions with the second and third reviewers resolved any uncertainty regarding eligibility.

### 2.3 Inclusion and exclusion criteria: articles and guidelines

The database‐identified articles were selected following specific inclusion and exclusion criteria. These included articles exploring the use of telemedicine for foot and ankle related patient consultations within a primary care setting. Furthermore, guidelines, practical guidance, consensus statements, expert opinions, case studies, editorials and narratives, and systematic reviews published in peer‐reviewed journals related to telemedicine for foot and ankle‐related patient consultations within a primary care setting were eligible for review. Any articles exploring the use of telemedicine for purposes other than patient consultations or which do not require direct interaction between patients and clinicians (such as in remote monitoring, training, health applications, and other forms of asynchronous communication) were excluded from this study. Moreover, articles exploring the use of telemedicine for lower limb wound management were also excluded. Articles where healthcare and medical students utilized telemedicine were excluded, as well as articles that did not fall under a primary care setting. Letters to the editor, patient education leaflets, and reviews that lack clear instructions on telemedicine for patient consultations were also excluded.

As for guidelines criteria that were followed, these included telemedicine practice guidelines produced by international professional bodies and relevant to the podiatry profession. However, telemedicine practice guidelines that fall beyond the scope of foot and ankle care management and guidelines produced by third parties that are not considered professional bodies were excluded from this study. All articles and guidelines that were used in this study had to be available in full text and in English.

### 2.4 Data extraction and synthesis

The principal researcher extracted data from the chosen abstracts and full texts, including information on the research country where the study was conducted, study design employed, outcomes of the study, function, target population, media, communication type, methodology, and limitations of these studies (Supporting Information: Appendix 3). Textual narrative synthesis^17^, ^18^ was used to chart the evidence into sectioned homogeneous research groupings based on the topics discussed or study methodologies. The principal n themes derived from this charting process were discussed among all reviewers for consensus. The levels of evidence and study types of the eligible studies synthesized to answer the research question were tabulated and can be found in Supporting Information: Appendix 4, which follows the one provided by Wright et al.,^19^ while indicators for study quality follow those mentioned in the research by Barske and Baumhauer^20^ which are available in Supporting Information: Appendix 4.

## 3 RESULTS

**Figure 1:**
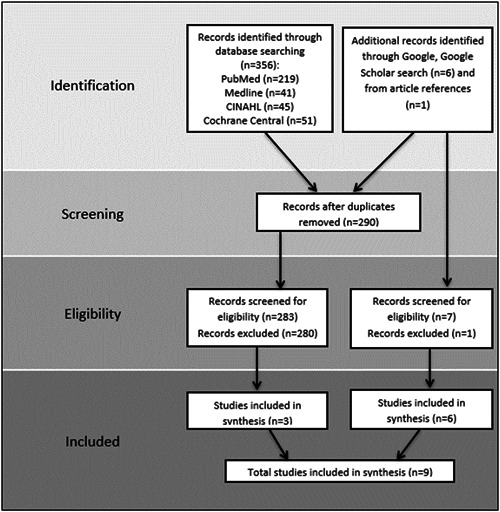
Flow diagram for selection of studies included in the scoping review

The search resulted in 356 articles; after removing duplicates, this number was reduced to 283. Six additional records were discovered via a Google Scholar search and one through an article reference search. After screening, six articles^21^, ^22^, ^23^, ^24^, ^25^, ^26^ and three institutional guidelines^27^, ^28^, ^29^ fitted the inclusion and exclusion criteria, which signaled paucity in the literature on this subject matter (Figure 1).

There were six eligible articles relating in whole or part to the foot and ankle.^21^, ^22^, ^23^, ^24^, ^25^, ^26^ However, the foot and ankle have been discussed from various professional viewpoints. For example, the only study that mentioned telemedicine in podiatry related to rheumatoid and musculoskeletal disease^24^; another three articles focused on the foot and ankle from an orthopedic standpoint.^21^, ^23^, ^26^ In contrast, two studies kept their virtual foot and ankle examinations open, referring to medical practitioners providing a virtual musculoskeletal examination.^22^, ^25^ Also, in the UK,^27^ Australia,^28^ and New Zealand,^29^ three different but overlapping sets of guidelines for telemedicine in podiatry were set up in 2020.

**Figure 2:**
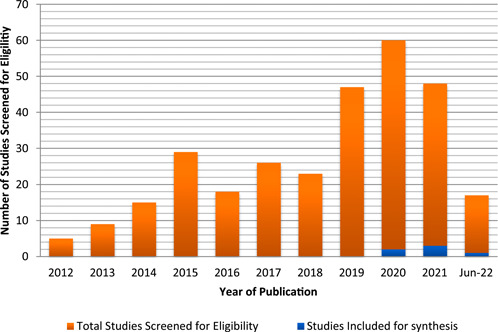
Search results generated by year of publication (excluding guidelines)

When articles were grouped by publication year (Figure 2), it was clear that interest in telemedicine research surged in 2020, when the worldwide coronavirus pandemic struck. The eligible studies were categorized based on the level of evidence and quality of research, the function of telemedicine, the type of media employed, the outcomes sought in that particular research and the presence of guidelines and recommendations provided.

### 3.1 Levels of evidence and quality of research

All eligible research articles were ranked according to their level of evidence, and the results were tabulated in Supporting Information: Appendix 4. Also, the quality of the research articles was investigated by looking at the research methods, analysis, and statistical indicators used in the studies that were part of this study, as shown in the table in Supporting Information: Appendix 4.

#### 3.1.1 The function of telemedicine and type of communication tools

**Figure 3:**
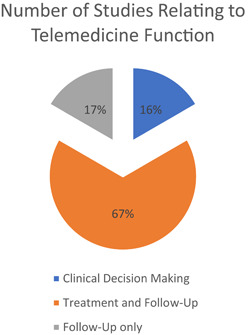
Percentage of studies identifying telemedicine function

Six research articles provided the primary functions of telemedicine. These were clinical decision‐making,^26^ treatment, and follow‐up^21^, ^22^, ^23^, ^25^ or only follow‐up,^24^ which are categorized below (Figure 3).

**Figure 4:**
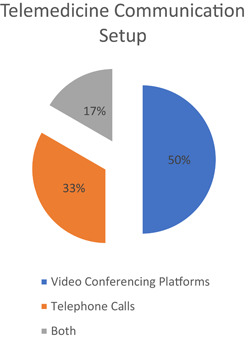
Chart showing percentages indicating telemedicine communication setup

All of the six identified research articles utilized synchronous communication.^21^, ^22^, ^23^, ^24^, ^25^, ^26^ Various configurations, including video conferencing,^21^, ^22^, ^23^ telephone calls,^24^, ^26^ or both,^25^ as depicted in the chart above, were used to achieve a telemedicine consultation (Figure 4). As for the recommended guidelines by public entities, they also referred to synchronous communication; but, they referred to telehealth only through video conferencing.^27^, ^28^, ^29^ Only guidelines by the College of Podiatry conceded to communication and social media apps such as WhatsApp and Telegram if no practical alternative was present and the benefits outweighed the risks.^27^

#### 3.1.2 Outcomes

**Figure 5:**
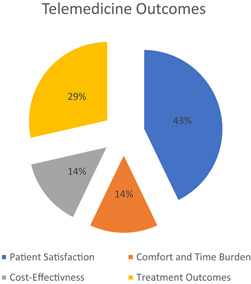
Charted percentages of telemedicine outcomes that resulted from the eligible studies

Four articles examined specific outcomes, which included patient satisfaction^23^, ^25^, ^26^; comfort, and time burden^23^; cost‐effectiveness^24^; and treatment outcomes.^24^, ^26^ Specifically, treatment outcomes were centered on the number of contact attempts required to reach a patient, call duration, and the number of completed calls per working day. Below is a chart comparing these results (Figure 5).

#### 3.1.3 Guidelines and recommendations

Four eligible research articles provided guidelines, protocols, or recommendations for foot and ankle‐related telemedicine practice. Eble et al.^21^ provided guidelines for patient attire and instructions for setting up the camera, which included recommended devices, patient clothing, examination space, patient position, and camera repositioning for standing and seated positions. In addition, they provided a comprehensive foot and ankle virtual examination template for clinicians that included a list of each examination to be performed, a checklist for medical record documentation, and corresponding verbal instructions to provide to patients during the virtual examination. While the study by Laskowski et al.^22^ provided similar patient and clinician recommendations but no set template, it focused primarily on the musculoskeletal examination of the entire body, with only a small segment addressing ankle examination.

In a separate study by Labib et al.,^25^ only the physician or a physician extender performing the telemedicine consultation was intended to receive the guidelines. The protocol outlined in this study was developed to provide consistent, high‐quality care to telemedicine patients. In addition, diagnosis‐directed examinations used in telemedicine visits to provide an objective examination of patients who cannot be physically examined are included. While some studies^21^, ^22^, ^25^ described the guidelines, the study by Sharma et al.^26^ suggested that an orthopedic clinical care pathway can introduce telemedicine consultations at different stages.

Furthermore, the College of Podiatry (UK),^27^ the Australian Podiatry Association,^28^ and the Podiatrists Board of New Zealand^29^ provided guidelines from an institutional standpoint. The purpose of these guidelines was primarily to clarify how to set up a telemedicine consultation, including the necessary patient setup. However, only the Australian Podiatry Association provided brief scenarios for when and how a telemedicine consultation for podiatry should be approached among the three guidelines.^28^ Also, all three institutional guidelines understood how necessary informed consent is and let patients decide for themselves if they wanted to be seen virtually.^27^, ^28^, ^29^

## 4 DISCUSSION

In general, practice guidelines on telemedicine with varying degrees of scope and depth have been made accessible, notably during the pandemic; however, there are no equivalent standards for podiatry. The given practice guidelines discussed in this study focus on the foot and ankle, but mainly from an orthopedic standpoint; nonetheless, most of these studies did not mention podiatrists' participation in telemedicine foot and ankle management. Podiatrists are the main protagonists in diagnosing, treating, and managing foot and ankle pathologies; hence, their input in the research and development of telemedicine practice guidelines relating to foot and ankle is imperative. The podiatrist's role overlaps with other allied and non‐allied health practitioners (physiotherapists, orthopedists, dermatologists, pedorthotists, etc.) due to the wide range of specializations in podiatric medicine. Standardized telemedicine guidelines must be established for podiatrists before they may be utilized in a primary care context by podiatrists and other foot and ankle‐related health practitioners.

The purpose of telemedicine guidelines is to provide practitioners with practical guidance for integrating telemedicine into current health systems.^30^ Leone et al. sought to identify telehealth practice guidelines for allied health professionals, including podiatrists. Gaps were identified in the practice guidelines, revealing commonalities and disparities with guidance from non‐allied health professionals.^31^ Moreover, the authors report that current guidelines do not adequately support allied health professionals to provide telehealth consultations.

The COVID‐19 pandemic necessitated the swift and immediate transition of patient care from traditional methods to the virtual concept; telemedicine was at the center of attention at the beginning and throughout the year 2020. Before the past 2 years, telemedicine for foot and ankle care, particularly podiatry in primary health care, was not investigated. Although Palmer et al.^24^ report that telephone appointments are beneficial in rheumatology podiatry practice, the authors of this review have since reconsidered their findings, due to developments in technology and communication. Clinicians today have access to many video‐based tools that would aid them in conducting telemedicine consultations, rendering telephone consultations a less preferred method of communication, particularly when visuals are needed.

Although telephone consultations are considered a telemedicine component, the information available to the practitioner assisting the patient is limited.^32^ Modern technology and video conferencing platforms can provide more in‐depth information, which, combined with other types of communication, can assist patients in receiving better care. However, this is dependent upon having the skills to utilize such gadgets and overcoming connectivity issues.^21^, ^22^, ^23^, ^25^

The articles and guidelines explored and compared in this review provide a sound basis for developing a new podiatry telemedicine model for the public primary care sector. Consideration should be given to tailoring the advice from the eligible publications in this evaluation towards foot and ankle care management. Expert opinion,^21^, ^22^, ^25^ a case series,^24^ a prospective cohort series,^26^ and a retrospective cohort series^23^ were the primary sources for the protocols and recommendations provided. Most studies used quantitative approaches like questionnaires with a cross‐section of retrospective and prospective participants. A control group was not included in any of the studies cited. Due to the lack of studies examining the use of telemedicine in foot and ankle care, their methods may be subject to debate. Therefore, there is a clear gap in the literature showing that these studies lack the methodological rigour needed to make a globally applicable telemedicine framework related to foot and ankle management.

Consequently, there is a need for more robust foot and ankle care research methodologies in this field. As for analysis, primary outcomes differed, but patient satisfaction was typically the primary outcome sought. Guidelines and recommendations are required to implement new care services. However, patient satisfaction is not the only outcome metric that must be considered to establish whether implementation has been successful or not. To evaluate the viability of a newly established service, it would be necessary to conduct additional research concentrating on feasibility with the aid of validated instruments that cover all the relevant variables. Typically, feasibility studies focus on several outcomes, including acceptability, demand, implementation, practicability, adaptability, integration, and expansion.^33^

Regarding the statistical aspect of assessing the level of evidence, only one research paper conducted a power analysis, displayed *p* values and standard deviations, and employed appropriate statistical analysis.^23^ Others restricted their statistical results to *p* values and appropriate statistical analysis,^25^, ^26^ Following the American Society of Plastic Surgeons' graded practice recommendations^34^, clinicians should consider all their options. Particularly by being flexible when making decisions, keeping an eye out for newly published evidence that clearly shows the balance of benefit to harm, and giving a patient choice about the type of care they want the most.

Active patient participation is imperative in healthcare decision‐making as it empowers patients and enhances service and health outcomes.^35^ Telemedicine has been employed for treatment and follow‐up situations, but traditional face‐to‐face consultations are still essential for patient care delivery and cannot be removed. Due to the nature of the podiatry profession, Stojmanovski^36^ stated a blended form of care (containing both in‐clinic and remote consultations), as this was mentioned and suggested by both podiatrists and patients. The ideal communication channel for podiatry telemedicine consultations has not yet been decided upon due to the novelty and originality of this study subject involving podiatry and telemedicine. Therefore, it is necessary to specify the fundamental justification for contemplating podiatric telemedicine for individual patients.

Depending on the patient's primary chief complaint, various treatment options may be available. In their study, Neville et al.^37^ did not provide practice guidelines for podiatric telemedicine practice. However, they provided an analysis that revealed podiatrists rated telemedicine as the most effective for prescribing medications and consulting about medical and dermatological issues. A previous study from the authors of the current work mentioned, podiatrists indicated telemedicine consultation would be most effective for preoperative purposes, followed by dermatological and postoperative concerns.^36^

Seamless introduction of telemedicine and service delivery is imperative to reassure patients that the same level of care as in‐clinic practice will be provided no matter what, to gain patient's trust and encourage patients to utilize telemedicine services. Any form of care delivery should always have the patient's welfare as its ultimate objective.

The demand for a service and the reason why such an innovative service is being proposed are crucial, and would later influence the implementation outcomes. According to Manz et al.,^23^ their study was the first to investigate the successful implementation of a new telemedicine model within an orthopedic foot and ankle division. Upon review of their research article, however, the successful implementation of this service was predicated solely on patient satisfaction, the convenience of service delivery, and the time burden. Furthermore, even though both orthopedic consultants and podiatrists focus on the foot and ankle, patients visit these healthcare providers for different reasons; therefore, successful implementation, in this case, has limitations, as the ailments treated by orthopedic consultants and podiatrists are not identical. Nevertheless, because orthopedic consultants and podiatrists are considered professions that require physical manipulation and intervention to assess patients, we are one step closer to comprehending telemedicine's role in foot and ankle‐related pathologies and care delivery.

As numerous studies have stated, telemedicine offers more accessible healthcare delivery and is, therefore, more practical, but how can the podiatry field adapt to this new care delivery method? Sharma et al.^26^ provided potential recommendations suggesting the use of telephone consultations in traditional in‐clinic practice as a follow‐up consultation or as a primary triage consultation in a more flexible pathway that is followed by an in‐person consultation before surgery. Hence, such recommendations in more general foot and ankle scenarios, particularly in podiatry, have not yet been provided. Therefore, such recommendations must be carefully considered and further evaluated among podiatric patients, as it is currently unclear whether patients with foot and ankle‐related conditions value this type of health service. Numerous healthcare professionals have implemented telemedicine to expand care. However, the full potential of telemedicine can only be realized through continuous public outreach and education, ensuring that all stakeholders are on board and the service is always evidence‐based.

### 4.1 Limitations

Despite efforts to thoroughly search the literature, some studies may have been overlooked. The limitations of this review were mainly related to the significant heterogeneity of the data among studies due to the different outcome measures used to assess telemedicine and the study cohorts included in these studies, which were mainly from an orthopedic clinical setting rather than podiatry. However, the methodological quality of the included studies was rigorously assessed, as seen in Supporting Information: Appendix 3.

## 5 CONCLUSION

This review identified a gap in the literature concerning foot and ankle telemedicine practice guidelines. Likewise, the authors suggest that standardized telemedicine practice guidelines for foot and ankle management be developed, with a particular focus on podiatrists and similar related professions, as they are currently non‐existent. There is also a great need for future research and collaboration between health professionals involved in foot and ankle pathologies. In addition, health institutions are encouraged to share guidelines to improve foot and ankle telemedicine consultations. Finally, developing universal rules for foot and ankle telemedicine practice can contribute to achieving one of the United Nations' 17 sustainable development goals by 2030, which are directly involved in promoting healthy lives and well‐being for people of all ages.

## AUTHOR CONTRIBUTIONS

**Lisa Ann Stojmanovski Mercieca**: Conceptualization; data curation; formal analysis; investigation; writing – original draft; writing – review & editing. **Cynthia Formosa**: Methodology; resources; supervision; writing – review & editing. **Nachiappan Chockalingam**: Methodology; writing – review & editing.

## CONFLICT OF INTEREST

The authors declare no conflict of interest.

## ETHICS STATEMENT

All the authors declare that the present work has been carried out in accordance with the Wiley's Best Practice Guidelines on Publishing Ethics and that is has been performed in an ethical and responsible way, with no research misconduct. The article has not been previously published and is not currently submitted elsewhere.

## TRANSPARENCY STATEMENT

The lead author Nachiappan Chockalingam affirms that this manuscript is an honest, accurate, and transparent account of the study being reported; that no important aspects of the study have been omitted; and that any discrepancies from the study as planned (and, if relevant, registered) have been explained.

## Supporting information

Appendix 1 – Scientific Database Research Strategy.

Appendix 2 – Google and Google Scholar Research Strategy.

Appendix 3 – Categorization of the data extracted.

Appendix 4 – Levels of evidence and quality of research.

Supporting information

## Data Availability

The data that support the findings of this study are available from the corresponding author upon reasonable request.

## References

[1] World Health Organization . *Implementing Telemedicine Services During Covid‐19: Guiding Principles And Considerations For A Stepwise Approach*. 2021. Accessed November 20, 2022. https://www.who.int/publications-detail-redirect/WPR-DSE-2020-032

[2] Kumar M , Rani P , Joshi B , Soni RK , Kumari A , Rohilla KK . Telemedicine as an unexpected catalyst during and beyond the COVID‐19 pandemic. Nepal J Epidemiol. 2022;12(1):1171‐1174. 35528453 10.3126/nje.v12i1.42459 PMC9057174

[3] Furlepa K , Tenderenda A , Kozłowski R , Marczak M , Wierzba W , Śliwczyński A . Recommendations for the development of telemedicine in Poland based on the analysis of barriers and selected telemedicine solutions. Int J Environ Res Public Health. 2022;19(3):1221. 35162248 10.3390/ijerph19031221 PMC8835106

[4] Kichloo A , Albosta M , Dettloff K , et al. Telemedicine, the current COVID‐19 pandemic and the future: a narrative review and perspectives moving forward in the USA. Fam Med Community health. 2020;8(3):e000530. 32816942 10.1136/fmch-2020-000530 PMC7437610

[5] Gajarawala SN , Pelkowski JN . Telehealth benefits and barriers. J Nurse Pract. 2021;17(2):218‐221. 33106751 10.1016/j.nurpra.2020.09.013 PMC7577680

[6] Snoswell CL , Chelberg G , De Guzman KR , et al. The clinical effectiveness of telehealth: a systematic review of meta‐analyses from 2010 to 2019. J Telemed Telecare. 2021:1357633X2110229. 10.1177/1357633X211022907 34184580

[7] Hazenberg CEVB , aan de Stegge WB , Van Baal SG , Moll FL , Bus SA . Telehealth and telemedicine applications for the diabetic foot: a systematic review. Diabetes Metab Res Rev. 2020;36(3):e3247. 31808288 10.1002/dmrr.3247 PMC7079242

[8] Tchero H , Noubou L , Becsangele B , Mukisi‐Mukaza M , Retali GR , Rusch E . Telemedicine in diabetic foot care: a systematic literature review of interventions and meta‐analysis of controlled trials. Int J Low Extrem Wounds. 2017;16(4):274‐283. 29168418 10.1177/1534734617739195

[9] Chen L , Cheng L , Gao W , Chen D , Wang C , Ran X . Telemedicine in chronic wound management: systematic review and meta‐analysis. JMIR Mhealth Uhealth. 2020;8(6):e15574. 32584259 10.2196/15574 PMC7381084

[10] Bondini CM , Sage S , Wilson BP , Hall MR , Wallis EAR . Modified telehealth for care of chronic wounds during the Coronavirus disease 2019 pandemic: a rapid literature review of alternative care modalities. Int Wound J. 2020;17(6):1960‐1967. 32902172 10.1111/iwj.13488 PMC7949008

[11] Bucki FM , Clay MB , Tobiczyk H , Green BN . Scoping review of telehealth for musculoskeletal disorders: applications for the COVID‐19 pandemic. J Manipulative Physiol Ther. 2021;44(7):558‐565. 35249750 10.1016/j.jmpt.2021.12.003 PMC8892222

[12] Wong B , Ward D , Gemmell K , et al. How is telehealth being utilized in the context of rehabilitation for lower limb musculoskeletal disorders: a scoping review. Physical Therapy Reviews. 2020;25(5‐6):350‐360.

[13] Gupta S , Sundaram SS . A review of telemedicine practice guidelines for COVID‐19 and elobal emergencies. Inquiry. 2022;59:00469580211059989. 35393886 10.1177/00469580211059989 PMC9251821

[14] Abbott LM , Miller R , Janda M , et al. A review of literature supporting the development of practice guidelines for teledermatology in Australia. Australas J Dermatol. 2020;61(2):e174‐e183. 32232852 10.1111/ajd.13249

[15] Tricco AC , Lillie E , Zarin W , et al. PRISMA extension for scoping reviews (PRISMA‐ScR): checklist and explanation. Ann Intern Med. 2018;169(7):467‐473. 30178033 10.7326/M18-0850

[16] Godin K , Stapleton J , Kirkpatrick SI , Hanning RM , Leatherdale ST . Applying systematic review search methods to the grey literature: a case study examining guidelines for school‐based breakfast programs in Canada. Syst Rev. 2015;4(1):138. 26494010 10.1186/s13643-015-0125-0 PMC4619264

[17] Kastner M , Tricco AC , Soobiah C , et al. What is the most appropriate knowledge synthesis method to conduct a review? Protocol for a scoping review. BMC Med Res Methodol. 2012;12:114. 22862833 10.1186/1471-2288-12-114 PMC3477082

[18] Lucas PJ , Baird J , Arai L , Law C , Roberts HM . Worked examples of alternative methods for the synthesis of qualitative and quantitative research in systematic reviews. BMC Med Res Methodol. 2007;7:4. 17224044 10.1186/1471-2288-7-4 PMC1783856

[19] Wright JG , Swiontkowski MF , Heckman JD . Introducing levels of evidence to the journal. J Bone Joint Surg Am. 2003;85(1):1‐3. 12533564

[20] Barske HL , Baumhauer J . Quality of research and level of evidence in foot and ankle publications. Foot Ankle Int. 2012;33(1):1‐6. 22381229 10.3113/FAI.2012.0001

[21] Eble SK , Hansen OB , Ellis SJ , Drakos MC . The virtual foot and ankle physical examination. Foot Ankle Int. 2020;41(8):1017‐1026. 32639852 10.1177/1071100720941020

[22] Laskowski ER , Johnson SE , Shelerud RA , et al. The telemedicine musculoskeletal examination. Mayo Clin Proc. 2020;95(8):1715‐1731. 32753146 10.1016/j.mayocp.2020.05.026 PMC7395661

[23] Manz WJ , Goel R , Fakunle OP , Labib SA , Bariteau JT . Feasibility of rapid development and deployment of a telemedicine program in a foot and ankle orthopedic practice. Foot Ankle Int. 2021;42(3):320‐328. 33040599 10.1177/1071100720963059

[24] Palmer JL , Siddle HJ , Redmond AC , Alcacer‐Pitarch B . Implementation of podiatry telephone appointments for people with rheumatic and musculoskeletal diseases. J Foot Ankle Res. 2021;14(1):4. 33413562 10.1186/s13047-020-00441-9 PMC7790049

[25] Labib SA , Goel R , Manz W , Bariteau J . Telemedicine foot and ankle visits in the COVID‐19 era. Foot Ankle Orthopaed. 2021;6(1):247301142199406. 10.1177/2473011421994068 PMC8702918 35097434

[26] Sharma A , Howgate D , Loizou C , et al. The application and patient‐reported experience of telephone consultations in elective foot and ankle orthopaedic surgery: 12‐month follow‐up. Foot Ankle Int. 2022;43(5):694‐702. 35081798 10.1177/10711007211068478

[27] The College of Podiatry . Guidance on remote consultations. 2020.

[28] Australian Podiatry Association . *Telehealth Consultations Guide for Podiatrists*. 2020. Accessed November 20, 2022. https://www.podiatry.org.au/documents/item/2229

[29] Podiatrists Board of New Zealand . *Telehealth Standards*. 2020. Accessed November 20, 2022. https://podiatristsboard.org.nz/wp-content/uploads/2020/04/Podiatrists-Board-Telehealth-Standards-April-2020.pdf

[30] Royal Australasian College of Physicians . *Telehealth Guidelines and Practical Tips*. Accessed November 20, 2022. https://www.racp.edu.au/docs/default-source/advocacy-library/telehealth-guidelines-and-practical-tips.pdf

[31] Leone E , Eddison N , Healy A , Royse C , Chockalingam N . Exploration of implementation, financial and technical considerations within allied health professional (AHP) telehealth consultation guidance: a scoping review including UK AHP professional bodies' guidance. BMJ Open. 2021;11(12):e055823. 10.1136/bmjopen-2021-055823 PMC8718347 34969656

[32] Hasani SA , Ghafri TA , Al Lawati H , et al. The use of telephone consultation in primary health care during COVID‐19 pandemic, Oman: perceptions from physicians. J Prim Care Community Health. 2020;11:2150132720976480. 33307943 10.1177/2150132720976480 PMC7739075

[33] Bowen DJ , Kreuter M , Spring B , et al. How we design feasibility studies. Am J Prev Med. 2009;36(5):452‐457. 19362699 10.1016/j.amepre.2009.02.002 PMC2859314

[34] Burns PB , Rohrich RJ , Chung KC . The levels of evidence and their role in evidence‐based medicine. Plast Reconstr Surg. 2011;128(1):305‐310. 21701348 10.1097/PRS.0b013e318219c171 PMC3124652

[35] Vahdat S , Hamzehgardeshi L , Hessam S , Hamzehgardeshi Z . Patient involvement in health care decision making: a review. Iran Red Crescent Med J. 2014;16(1):e12454. 24719703 10.5812/ircmj.12454 PMC3964421

[36] Stojmanovski Mercieca LA . *Podiatric Telemedicine: An Evidence‐Based Approach* (Masters). University of Malta. 2021. Accessed November 20, 2022. https://www.um.edu.mt/library/oar/handle/123456789/85647

[37] Neville K , Black AT , Fridman R . Epidemiological survey of the impact of COVID‐19 on telemedicine in the practice of foot and ankle surgery in the United States. J Foot Ankle Surg. 2021;60(3):455‐460. 33518507 10.1053/j.jfas.2020.08.003 PMC7581410

