# Supplementary material for: A scoping review of foot and ankle telemedicine guidelines
Source: Health Sci Rep. 2023 Jan 20;6(1):e1076. doi: 10.1002/hsr2.1076 (PMC9860371; doi:10.1002/hsr2.1076)
Supplement: Supplementary file 1 — Appendix 1 – Scientific Database Research Strategy. [file HSR2-6-e1076-s002.pdf]

PubMed search strategy		
Initial search yielded 349 results<br>Filters applied<br>▪ Publication date within the last 10 years – 275 results<br>▪ Articles involving only humans – 221 results<br>▪ Articles in the English language only - 219		
Search term	MeSH heading	Free text
Telemedicine	Telehealth<br>Remote Consultation	(Telehealth OR tele-health OR telemedicine or Tele-medicine OR telecare OR tele-care OR telehomecare OR tele-homecare OR<br>telehealthcare OR tele-healthcare OR teleconsultation OR tele-consultation OR “video consultation” OR video-consultation OR<br>videoconference OR video-conference OR “video conferencing” OR teleconference OR tele-conference OR telecommunication OR<br>tele-communication OR tele-monitoring OR telemonitoring OR telerehab* OR tele-rehab* OR telecoaching OR tele-coaching OR<br>ehealth OR e-health OR “electronic health” OR ecare OR e-care OR “electronic care” OR “digital health” OR mhealth OR m-health<br>OR “mobile health” OR etherapy OR e-therapy OR “electronic therapy”)<br><br>(online OR on-line OR virtual OR internet-based OR web OR web-based OR remote OR distant OR electronic OR instant OR<br>telematic OR interactive OR telephone OR video OR mobile OR phone OR internet OR computer OR “computer assisted” OR<br>computer-assisted OR “computer aided” OR computer-aided) AND (rehab* OR therapy OR treatment OR care OR consultation OR<br>counselling OR monitoring OR surveillance OR communication)
Guidelines	Guidelines	(guideline* OR guidance OR standard* OR pathway* OR protocol* OR advisor* OR summar* OR polic* OR statement* OR<br>blueprint* OR regulation* OR circular* OR recommendation* OR dissemination* OR priority OR reform OR plan OR program* OR<br>agenda OR “declaration”)
Foot health	Podiatry	(feet OR foot OR podiatr* OR “foot health” OR chiropody* OR “foot care” OR “foot bones” OR “foot

		joints" OR "foot injuries" OR "foot deformities" OR "ankle injuries" OR "Achilles tendon" OR "musculoskeletal lower limb" OR "foot disease" OR "foot infection")

CINAHL (EBSCOhost) search strategy		
Initial search yielded 150 results<br>Filters applied<br>▪ Publication date within the last 10 years – 104 results<br>▪ Articles involving only humans – 46 results<br>▪ Articles in the English language only - 45		
Search term	MeSH heading	Free text
Telemedicine	Telehealth<br>Remote Consultation	(Telehealth OR tele-health OR telemedicine or Tele-medicine OR telecare OR tele-care OR telehomecare OR tele-homecare OR<br>telehealthcare OR tele-healthcare OR teleconsultation OR tele-consultation OR "video consultation" OR video-consultation OR<br>videoconference OR video-conference OR "video conferencing" OR teleconference OR tele-conference OR telecommunication OR<br>tele-communication OR tele-monitoring OR telemonitoring OR telerehab* OR tele-rehab* OR telecoaching OR tele-coaching OR<br>ehealth OR e-health OR "electronic health" OR ecare OR e-care OR "electronic care" OR "digital health" OR mhealth OR m-health<br>OR "mobile health" OR etherapy OR e-therapy OR "electronic therapy")<br><br>(online OR on-line OR virtual OR internet-based OR web OR web-based OR remote OR distant OR electronic OR instant OR<br>telematic OR interactive OR telephone OR video OR mobile OR phone OR internet OR computer OR "computer assisted" OR<br>computer-assisted OR "computer aided" OR computer-aided) AND (rehab* OR therapy OR treatment OR care OR consultation OR<br>counselling OR monitoring OR surveillance OR communication)
Guidelines	Guidelines	(guideline* OR guidance OR standard* OR pathway* OR protocol* OR advisor* OR summar* OR polic* OR statement* OR

		blueprint* OR regulation* OR circular* OR recommendation* OR dissemination* OR priority OR reform OR plan OR program* OR agenda OR “declaration”)
Foot health	Podiatry	(feet OR foot OR podiatr* OR “foot health” OR chiropody* OR “foot care” OR “foot bones” OR “foot joints” OR “foot injuries” OR “foot deformities” OR “ankle injuries” OR “Achilles tendon” OR “musculoskeletal lower limb” OR “foot disease” OR “foot infection”)

Cochrane Central Register of Controlled Trials (EBSCOhost) search strategy		
Initial search yielded 88 results<br>Filters applied<br>▪ Publication date within the last 10 years – 79 results<br>▪ Articles in the English language only - 51		
Search term	MeSH heading	Free text
Telemedicine	Telehealth<br>Remote Consultation	(Telehealth OR tele-health OR telemedicine or Tele-medicine OR telecare OR tele-care OR telehomecare OR tele-homecare OR telehealthcare OR tele-healthcare OR teleconsultation OR tele-consultation OR “video consultation” OR video-consultation OR videoconference OR video-conference OR “video conferencing” OR teleconference OR tele-conference OR telecommunication OR tele-communication OR tele-monitoring OR telemonitoring OR telerehab* OR tele-rehab* OR telecoaching OR tele-coaching OR ehealth OR e-health OR “electronic health” OR ecare OR e-care OR “electronic care” OR “digital health” OR mhealth OR m-health OR “mobile health” OR etherapy OR e-therapy OR “electronic therapy”)<br><br>(online OR on-line OR virtual OR internet-based OR web OR web-based OR remote OR distant OR electronic OR instant OR telematic OR interactive OR telephone OR video OR mobile OR phone OR internet OR computer OR “computer assisted” OR computer-assisted OR “computer aided” OR computer-aided) AND (rehab* OR therapy OR treatment OR care OR consultation OR

		counselling OR monitoring OR surveillance OR communication)
Guidelines	Guidelines	(guideline* OR guidance OR standard* OR pathway* OR protocol* OR advisor* OR summar* OR polic* OR statement* OR<br>blueprint* OR regulation* OR circular* OR recommendation* OR dissemination* OR priority OR reform OR plan OR program* OR<br>agenda OR "declaration")
Foot health	Podiatry	(feet OR foot OR podiatr* OR "foot health" OR chiropody* OR "foot care" OR "foot bones" OR "foot joints" OR "foot injuries" OR "foot deformities" OR "ankle injuries" OR "Achilles tendon" OR "musculoskeletal lower limb" OR "foot disease" OR "foot infection")

Medline search strategy		
Initial search yielded 71 results<br>Filters applied<br>▪ Publication date within the last 10 years – 56 results<br>▪ Articles involving only humans – 41 results<br>▪ Articles in the English language only - 41		
Search term	MeSH heading	Free text
Telemedicine	Telehealth<br>Remote Consultation	(Telehealth OR tele-health OR telemedicine or Tele-medicine OR telecare OR tele-care OR telehomecare OR tele-homecare OR<br>telehealthcare OR tele-healthcare OR teleconsultation OR tele-consultation OR "video consultation" OR video-consultation OR<br>videoconference OR video-conference OR "video conferencing" OR teleconference OR tele-conference OR telecommunication OR<br>tele-communication OR tele-monitoring OR telemonitoring OR telerehab* OR tele-rehab* OR telecoaching OR tele-coaching OR<br>ehealth OR e-health OR "electronic health" OR ecare OR e-care OR "electronic care" OR "digital health" OR mhealth OR m-health<br>OR "mobile health" OR etherapy OR e-therapy OR "electronic therapy")<br><br>(online OR on-line OR virtual OR internet-based OR web OR web-based OR remote OR distant OR electronic OR instant OR

		telematic OR interactive OR telephone OR video OR mobile OR phone OR internet OR computer OR "computer assisted" OR computer-assisted OR "computer aided" OR computer-aided) AND (rehab* OR therapy OR treatment OR care OR consultation OR counselling OR monitoring OR surveillance OR communication)
Guidelines	Guidelines	(guideline* OR guidance OR standard* OR pathway* OR protocol* OR advisor* OR summar* OR polic* OR statement* OR blueprint* OR regulation* OR circular* OR recommendation* OR dissemination* OR priority OR reform OR plan OR program* OR agenda OR "declaration")
Foot health	Podiatry	(feet OR foot OR podiatr* OR "foot health" OR chiropody* OR "foot care" OR "foot bones" OR "foot joints" OR "foot injuries" OR "foot deformities" OR "ankle injuries" OR "Achilles tendon" OR "musculoskeletal lower limb" OR "foot disease" OR "foot infection")
