# Supplementary material for: A scoping review of foot and ankle telemedicine guidelines
Source: Health Sci Rep. 2023 Jan 20;6(1):e1076. doi: 10.1002/hsr2.1076 (PMC9860371; doi:10.1002/hsr2.1076)
Supplement: Supplementary file 2 — Appendix 2 – Google and Google Scholar Research Strategy. [file HSR2-6-e1076-s005.pdf]

Custom Google Scholar Search Engine for Foot and Ankle Telemedicine Practice Guidelines
Note: filter were applied.
Date searched: 5th May 2022

#	Search	# results	# results screened	# new potentially relevant records
1	telemedicine AND guidelines AND foot health	21500	50	0
2	telemedicine OR telehealth OR ehealth AND guidelines AND foot health	26800	50	0
4	telemedicine AND guidelines AND foot disease OR foot care	24000	50	4
5	telemedicine AND podiatry AND practice guidelines	2560	50	0

Custom Google Search Engine for Foot and Ankle Telemedicine Practice Guidelines
Note: filter were applied.
Date searched: 5th May 2022

#	Search	# results	# results screened	# new potentially relevant records
1	telemedicine AND guidelines AND foot health	1030000	50	0
2	telemedicine OR telehealth OR ehealth AND guidelines AND foot health	16300000	50	0
4	telemedicine AND guidelines AND foot disease OR foot care	17000000	50	0
5	telemedicine AND podiatry AND practice guidelines	119000	50	2
